# Antibacterial Activity of Ciprofloxacin-Based Carbon Dot@Silver Nanoparticle Composites

**DOI:** 10.1021/acsomega.5c00142

**Published:** 2025-03-14

**Authors:** Paloma Maria de Sousa Araujo, Milena Lima Guimarães, André Rossi, Mateus Matiuzzi da Costa, Leonardo De Boni, Helinando Pequeno de Oliveira

**Affiliations:** † Instituto de Pesquisa em Ciência dos Materiais, Universidade Federal do Vale do São Francisco, 48902-300 Juazeiro, BA, Brazil; ‡ RENORBIO - Northeast Biotechnology Network, Universidade Federal Rural de Pernambuco (UFRPE), 52171-900 Recife, Pernambuco, Brazil; § Centro Brasileiro de Pesquisa Física (CBPF), 22290-180 Rio de Janeiro, RJ, Brazil; ∥ Instituto de Física, Universidade de São Paulo, 13566-590 São Carlos, SP, Brazil

## Abstract

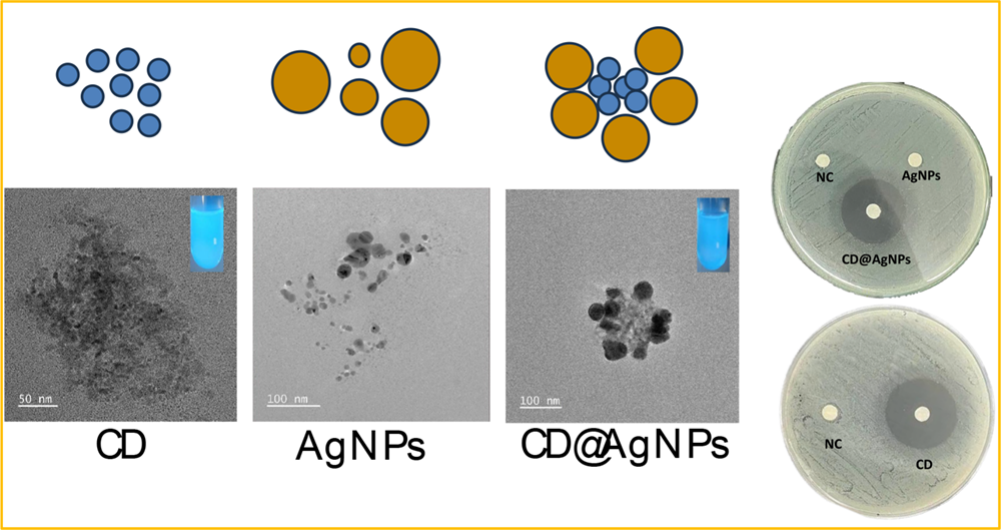

The combined green synthesis of carbon dots (CDs) from the hydrothermal conversion of ciprofloxacin and silver nanoparticles (AgNPs) using sodium alginate as a reducing and stabilizing agent results in arrangements of nanostructures (CD@AgNP composites) with positive surface charge that electrostatically interact with Gram-positive and Gram-negative bacteria in the planktonic form and also biofilm forms, inhibiting their growth and adhesion on surfaces. Outstanding performance for CD-based materials results in a 5-log reduction in colony-forming units (CFU/mL) of *E. coli* after 1 h of treatment and a decrease of 99.32% in the consolidated biofilm of *S. aureus*. These nanostructures result in the intrinsic fluorescence of CDs and an overall eco-friendly preparation process that can be explored in disinfection procedures based on the direct administration of a sanitizer based on nanoparticles dispersed in an aqueous solution. This process is justified by the adequate conversion of antibiotics in positively charged CDs and composites with AgNPs, resulting in nanocomposites in which the prevailing cationic effect facilitates their incorporation and diffusion into bacterial membrane cells.

## Introduction

Antibiotics have been extensively applied in controlling human infections and livestock production, providing residues of active molecules in animal body issues (meat, milk, and eggs).^1^ The disposal of expired antibiotics into the environment represents another critical source for the contamination of groundwater, soil, and air,^2^ which, in addition to the untreated wastewater^3^ (contaminated by fecal pollution and hospital residues),^4^ propagates antibiotic-resistant bacteria genes,^5^ favoring the gene mutation mechanisms and the uptake of genetic components in the bacterial genome from efflux pumps, modifying the bacterial outer membrane.^6^

Consequently, bacterial adaptive mechanisms favor multidrug resistance (MDR) due to the overuse of antibiotics. This process reduces the susceptibility of microorganisms to medicines, with antibiotic resistance particularly dangerous for immunocompromised patients.^7,8^ A group of bacteria known as ESKAPE (*Enterococcus faecium*, *Staphylococcus aureus*, *Klebsiella pneumoniae*, *Acinetobacter baumannii*, *Pseudomonas aeruginosa*, and Enterobacter spp.)^9^ has been associated with high morbidity and mortality in hospitalized patients,^10,11^ representing a critical issue that needs to be addressed with new alternatives to antibiotic-based treatment.^9^

The assembly of nanostructures has been considered for a broad range of applications, including the electrochemical detection of analytes from metal–organic frameworks decorated with silver nanoparticles (AgNPs),^12^ the antibacterial activity of hollow-dodecahedra graphene oxide-cuprous oxide for application in a broad spectrum of pathogenic bacteria,^13^ and nanostructures based on soft robots (nanoaggregates) that are magnetically guided toward targeted infections, controlled by the use of magnetocaloric effect under a high-frequency alternating field in the gastrointestinal tract.^14^ In particular, the production of metal nanoparticles with antibacterial activity has attracted attention in the literature due to their superior advantages, such as high surface area, quantum confinement, and UV–vis emissive properties, which facilitate the light-induced activity of nanoparticles in modulating their permeability through the bacterial cell wall^15^ against biofilms of ESKAPE pathogens.^16^

Moreover, metal nanoparticles (such as AgNPs)^17^ have been successfully applied as antibacterial agents with activity based on releasing ions, inducing reactive oxygen species (ROS), electrostatic interaction with bacterial cells, and metal-ion homeostasis that results from the strong ability of these materials to bind with N, O, and S-based groups, which are characteristic of organic compounds.^18^

Alternatively, carbon quantum dots (CD)-zero-dimensional carbon sources^19^ with a diameter in the 2–20 nm range^20^ can be produced by several green synthesis methods^21^ such as converting natural sources by hydrothermal treatment and using benign solvents at moderate synthesis temperatures (120–240 °C). In addition to these eco-friendly properties, the abundance of carbon sources (such as residues of agriculture) favors the production of nanomaterials with size-dependent properties for use in bioimaging-based applications^20^ owing to their typical absorbance in the range of 230–270 nm,^19^ the quasi-spherical shape of nanoparticles, high quantum yield, and water solubility,^20^ which are enhanced by the active sites for light absorption.^22^

In addition, the antibacterial activity of CDs results from the ROS generation profile at a level superior to the bacterial antioxidant defense, producing favorable results that lead to bacterial death, attributed to the doping level induced by heteroatoms and their high density of free electrons.^23^

Fresh or expired medicines, such as ciprofloxacin, can be considered a relevant source for carbon dot (CD) production.^24^ Ciprofloxacin is a third-generation quinolone antibiotic that proved to be inefficient against several bacteria (MDR processes). Miao et al.^25^ reported the conversion of ciprofloxacin into CD for antibacterial agent production (pure species and complexes of CDs with Cu-based structures).

Herein, the preparation of green composites of AgNPs and CDs by the hydrothermal treatment with sodium alginate (for AgNP reduction) and ciprofloxacin (source for CD production) is proposed as components of the fluorescent species with antibacterial activity against Gram-positive and Gram-negative bacteria evaluated from agar diffusion experiments, time-kill assays, and antibiofilm activity assays, with promising results for application as additives for sanitizing agents.

## Materials and Methods

### Materials

Acetic acid (Vetec, 99.7%), silver nitrate (Sigma-Aldrich, 99%), fresh ciprofloxacin (Sigma-Aldrich, 98%), sodium citrate (Sigma-Aldrich, 99%), sodium alginate (Sigma-Aldrich), silicone sheets (Neves & Neves), brain heart infusion medium (BHI, Kasvi), plate count agar medium (PCA, Kasvi), tryptic soy broth (TSB, Kasvi) and bacteriological agar (ACS Científica) were of analytical grade and used as received.

### Synthesis of CDs

CDs were synthesized according to the methodology of Miao et al.,^25^ described as follows: 10 mg of ciprofloxacin is dissolved into 5 mL of acetic acid and dispersed in 25 mL of ultrapure water (18.2 MΩcm) being treated in an ultrasonic bath for 10 min. Then, the solution is disposed of in a PTFE reactor (Bench Top Reactor System) and kept in an oven at 180 °C for 8 h. After this step, the solution is cooled to room temperature and dialyzed for 2 h through a dialysis bag (MW 3500), in which the retained solution and residues that crossed the membrane were stored in a refrigerator for characterization.

### Hydrothermal Synthesis of AgNPs and Complexes of CDs/AgNPs

The hydrothermal synthesis of AgNPs was conducted according to the procedure reported by Pan et al.^26^ by the use of sodium alginate as a reducing agent. In a standard procedure, 1.0 mL of AgNO_3_ (0.04 M) and 1.6 mL of 0.5 w/v% of Na-Alg in 32 mL of water were mixed and transferred to a stainless-steel autoclave with a PTFE reactor and heated to 100 °C for 6 h. Then, the resulting solution was cooled to ambient temperature and stored. The synthesis of CDs/silver nanoparticles (CD@AgNPs) composites was conducted as described in Section 2.2 with some modifications, as follows.

10 mg of ciprofloxacin is dissolved into 5 mL of acetic acid, dispersed in 25 mL of as-prepared solution of AgNPs, and treated in an ultrasonic bath for 10 min. Then, the solution is disposed of in a PTFE reactor (Bench Top Reactor System) and kept in an oven at 180 °C for 8 h. After this step, the solution is cooled to room temperature and dialyzed for 2 h through a dialysis bag (*M*_W_ 3500), in which the retained solution was stored in a refrigerator.

### Characterization Techniques

The absorbance spectra of AgNPs, CD, and CD@AgNP aqueous solutions were acquired in a Hach DR5000 single-beam UV–vis spectrophotometer from 200 to 800 nm with a step of 1 nm.

The nanoparticles’ surface potential and size distribution were determined using a Malvern Zetasizer nanoparticle analyzer, with the measurement conducted at room temperature. The fluorescence quantum yield was determined using Brower’s method^27,28^ with stilbene dissolved in methanol as a reference sample (ϕ_f_ = 95%).^29^ A UV–vis spectrophotometer (Shimadzu UV-1800) and a fluorimeter (Hitachi F7000 fluorimeter) were used to measure the absorption and fluorescence of the samples dissolved in water. The samples were kept in a 10 mm quartz cuvette with a low molar concentration, keeping the absorbance at the excitation wavelength (300 nm) at ca. 0.2 to avoid the sample’s fluorescence reabsorption. Excited samples obtained a fluorescence lifetime at 300 nm with a 190 fs pulsed laser.

The fluorescence signal was collected by a fast silicon photodetector with an increased time of about 700 ps and analyzed by using a GHz digital oscilloscope. X-ray diffraction (XRD) patterns of the nanoparticles and the corresponding composite were provided by a Miniflex powder diffractometer (Rigaku) equipped with a Cu-Kα radiation source (λ = 1.5406 Å), operating at 40 kV and 15 mA with data collected over a 2θ range of 20°–90° and a scan speed of 10°/min at a step of 0.02° at room temperature.

The FTIR spectrum was performed on a Shimadzu IRPrestige-21 Fourier transform infrared spectrometer exploring the KBr method. Transmission electron microscopy (TEM) images for nanoparticles were performed in a JEOL 2100F instrument operated at an accelerating voltage of 200 kV and equipped with a One View 16 Mega Pixel CMOS digital camera (GATAN).

### Antibacterial Activity Evaluation

#### Determination of Minimum Inhibitory Concentration (MIC) and Minimum Bactericidal Concentration (MBC)

All antibacterial assays were carried out with the strains *S. aureus* (ATCC 25923) and *E. coli* (ATCC 25922). The broth microdilution method determined the MIC of the nanostructures (AgNPs, CD, and CD@AgNPs). With this aim, the 96-well microtiter plates were partially filled with 100 μL of trypticase soy broth that received in the following step 100 μL of the as-prepared solution of nanoparticles (AgNPs, CD, and CD@AgNPs) that were successively diluted from the mother solution ranging from 1:1 until 1:128. After this step, the bacterial inoculum was prepared by transferring a batch of isolated colonies to a test tube containing 5 mL of saline solution. The turbidity of the bacterial suspension was verified at 0.5 on the McFarland scale (10^8^ CFU/mL). Following a dilution of 1:100, the concentration was reduced to 10^6^ CFU/mL, and 10 μL was transferred to the microplates in all wells containing the antibacterial solutions for positive controls. For each well, aliquots of 2,3,5-triphenyltetrazolium chloride (30 μL at 0.1% w/v) and, after that, incubation for 30 min at 37 °C were conducted. Experiments were performed in triplicate. After that, the visual inspection of the wells identified a pink coloration, indicating bacterial growth. The MIC value of nanoparticles corresponds to the minimum concentration at which no color change is observed in a well.

For MBC determination, instead of 30 min, the wells were incubated for 24 h at 37 °C. After the incubation, the contents of the microplates were inoculated into a Petri dish G, containing BHI for *S. aureus* and PCA for *E. coli*, with the aid of a replicator and incubated at 37 °C for 24 h. The results were observed after the incubation time, from the growth of colonies on the plate, with the MBC considered the lowest concentration able to kill the bacteria.

### Agar Diffusion Assays

The agar diffusion test was carried out with *S. aureus* and *E. coli* strains at 1 × 10^8^ CFU/mL, determined according to the previously discussed method. For evaluation of the diffusion of species, a fixed content of the as-prepared nanoparticles (with 5 μL) was dispersed in a circular disk of filter paper (5 mm in diameter), allowing natural evaporation to take place. Dried disks impregnated with the nanoparticles were carefully deposited on culture media (TSB+Agar) in the presence of the bacteria under test.

Each plate containing the culture media and bacteria in the presence of disks impregnated with each antibacterial agent was incubated at 37 °C for 24 h. The direct visual inspection of plates after the incubation allows the direct determination of the halo, the corresponding size of the growth inhibition zone, and a direct estimation of the antibacterial activity of the released material.

### Time-Kill Kinetics Assays

As a first step, the bacterial suspension was prepared by transferring a portion of the colonies to tubes containing 5 mL of saline solution and comparing it to 0.5 on the McFarland scale (10^8^ CFU/mL). Then, 1 mL was transferred to a tube containing 9 mL of saline solution to obtain 10^7^ CFU/mL. Then, 1 mL was transferred to two tubes containing 9 mL of TSB broth, providing a concentration of 1 × 10^6^ CFU/mL.

The time-kill kinetics assay was carried out with bacterial suspensions of the *S. aureus* and *E. coli* strains at 1 × 10^6^ CFU/mL concentrations. The kinetics were evaluated over a range of 6 h, in which for every hour of treatment, aliquots of 100 μL of each sample were removed for plating using the pour-plate method by dispersing the solution in a Petri dish and adding 20 mL of BHI for *S. aureus* and PCA for *E. coli* at 45 °C. Then, the plates were incubated at 37 °C for 24 h. After this process, viable colony counts were carried out on each plate as a function of the time of contact with the antibacterial agent. Experiments were conducted in triplicate for each experimental system (AgNPs, CD, and CD@AgNPs).

### Antibiofilm Assays

For the biofilm eradication capacity evaluation, the methodology reported by Azevedo et al.^30^ was conducted as follows: silicone-based supports were cut in squares of (1 × 1) cm^2^ for the following growth of *S. aureus* biofilm and the evaluation of the influence of nanomaterials dispersed in water on the consolidated biofilm. With this aim, six silicone supports were arranged in a 24-well plate, with three wells corresponding to the positive control conditions (no treatment) and the remaining wells corresponding to different treatments (AgNPs, CD, and CD@AgNPs).

Then, 1.5 mL of the bacterial suspension at 1 × 10^6^ CFU/mL *S. aureus* was disposed of in the six wells, and the others were filled with a saline solution and incubated at 37 °C for 24 h. After incubation, the culture medium was removed from the wells, and the silicone supports were washed three times with a saline solution. Then, the supports were transferred to tubes containing 5 mL of a saline solution. Each tube was vortexed three times for 30 s, followed by an additional 30 s under sonication to complete the detachment of the biofilm from the silicone surface.

Serial dilution was performed using seven Eppendorf tubes, filled with 900 μL of saline solution. 1000 μL of the contents of the tube with the detached biofilm was transferred to the first empty tube, and 100 μL was transferred to the next one containing 900 μL of saline. Following this sequence, successive dilutions were provided: 1:10^0^ , 1:10^1^, 1:10^2^, 1:10^3^, 1:10^4^, 1:10^5^, and 1:10^6^, and the plating was carried out on plates with a TSA medium by disposal of 30 μL of each experimental system.

## Results and Discussion

**Figure 1:**
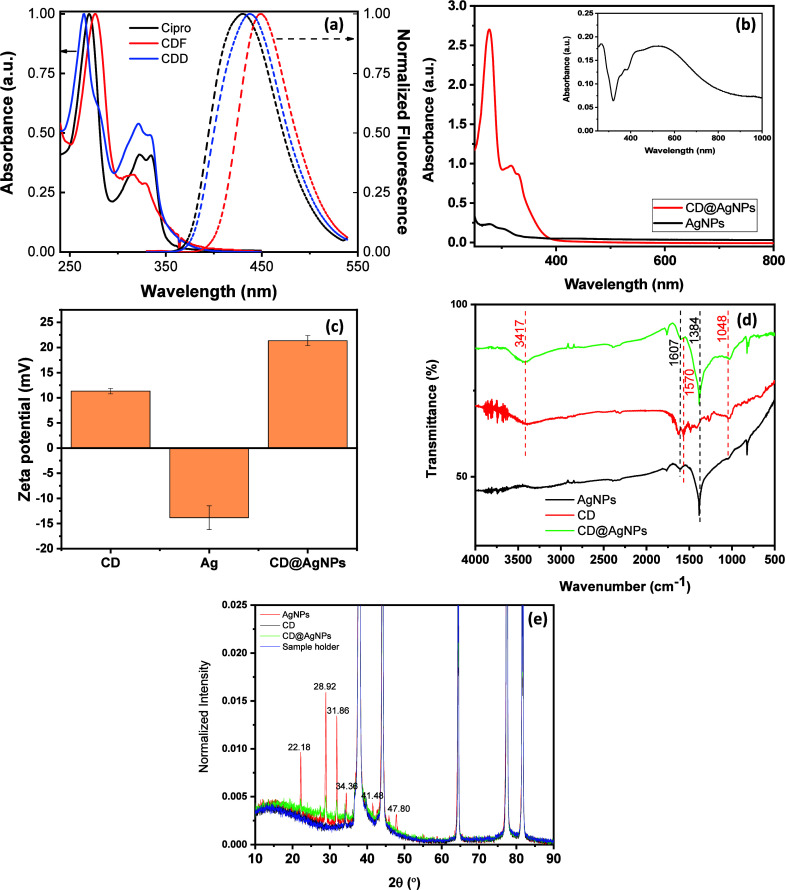
(a) Absorbance (solid lines) and emission (dashed lines) for the pure antibiotic solution (ciprofloxacin) (in black), retained nanostructures in a dialysis bag (CDD) (blue lines), and filtered residues (CDF) (red lines). (b) Absorbance of AgNP and CD@AgNP nanoparticles. (c) Comparison of the ZP of synthesized nanoparticles (CD, AgNPs, CD@AgNPs). (d) FTIR spectra of nanoparticles of the CD (curve in red), AgNPs (curve in black), and CD@AgNPs (curve in green). (e) Experimental XRD patterns for CD, CD@AgNPs, and AgNPs in comparison with the sample holder response.

The absorbance in the UV–vis region and the emission of as-prepared CDs were evaluated under two conditions (converted CDs retained into the dialysis bag—CDD and filtered products that passed through the dialysis bag—CDF) for comparison with the response of the ciprofloxacin solution (source for CD production). As shown in Figure 1a, three prominent peaks are observed in the absorbance spectrum for the ciprofloxacin solution at 270, 322, and 335 nm. In correspondence, these three characteristic peaks are also observed for converted species (CDD and CDF).

The first peak of the CDD is blue-shifted to 264 nm, while CDF’s characteristic peak is shifted to 277 nm. According to the literature,^25^ the strong absorption peak at 275 nm for CDs has been attributed to the aromatic sp^2^ structural domain (C=C, C–C), with the peak around 325 nm attributed to the C=O and C–N bonds, an indication that the ring structure of ciprofloxacin has also been observed in converted CDs. The emission of pure ciprofloxacin is centered at 431 nm, with a red shift in the emission of the converted species at 438 nm for CDD and 450 nm for the CDF species.

Regarding the fluorescence, the measured value for the quantum yield of the ciprofloxacin solution is 19%. The corresponding values for the dialyzed nanoparticles were 17% (CDF). On the other hand, an increase in the quantum yield to 26% is observed for converted CDs (inside the dialysis bag), indicating a direct relationship with the successful conversion rate of antibiotics into the highly fluorescent species (CDs).

The absorbance of AgNPs is characterized by a broad peak centered at 519 nm (red-shifted from the typical plasmon band of AgNPs), see Figure 1b, assigned to the formation of aggregates with size in the order of 90–100 nm.^31^ Strong absorbance in the UV region attributed to the CD nanoparticles is also observed for CD@AgNP composites, which prevail in the overall spectrum (Figure 1b in red).

In addition to the optical response, another critical parameter to be considered in the characterization of nanoparticles is the zeta potential (ZP) of the resulting material since it preserves a relationship not only with the stability of nanoparticles in solution but also in terms of the surface charge in nanostructures.

ZP analysis of nanostructures is shown in Figure 1c, and it indicated that CD nanoparticles carry positive charges (+11.33 mV), in agreement with those reported by Miao et al.^25^ for the corresponding experimental systems as a consequence of the surface charge for the converted species from ciprofloxacin. On the other hand, sodium alginate acts as a reducer and stabilizer for synthesizing AgNPs. Due to the high density of carboxyl and hydroxyl groups along their backbone, a high negative charge density captures Ag^+^ by the strong interaction between AgNPs and the oxygen from species in the polysaccharide chains.^32^

As a result, the negative ZP obtained for the pure AgNP solution reduced by Na-Alg is negative (−13.83 mV), which is attributed to the prevailing negative charge (carboxyl and hydroxyl groups of the net) in which AgNPs are embedded. Consequently, the prevailing electrostatic interaction of CDs and AgNPs favors the assembly of components into the composite CD@AgNPs that presented a prevailing positive charge (+21.37 mV), representing a critical factor underpinning the electrostatic interaction of the composite with bacterial cell walls.

The FTIR spectra of CD, AgNPs, and CD@AgNPs nanoparticles are summarized in Figure 1d. Characteristic peaks of CDs converted from ciprofloxacin are in agreement with those reported by Miao et al.,^25^ as follows: the stretching vibration of the −OH bond is observed at 3417 cm^–1^, while bands at 1048 and 1570 cm^–1^ are assigned to stretching vibrations of the C–O bond and the C=O bond, respectively, confirming the disposition of carboxyl (−COOH) groups on CDs.

Regarding the AgNPs, characteristic bands at 1607 cm^–1^ are assigned to asymmetric and symmetric vibrations of carboxylate with characteristic bands for C–C and C–N stretching observed at 1384 cm^–1^.^33^ An important aspect to be considered is the C–O–C stretching vibration from alginate^34^ confirming the adsorption of the two main functional groups (−COO and C–O–C) on the AgNP structures.^35^

XRD patterns of nanoparticles are provided in Figure 1e, in which it is possible to observe the superposition of characteristic peaks of AgNPs with the aluminum peaks' response of the sample holder at 38.1^ο^, 44.32^ο^, 64.43^ο^, and 77.41^ο^.^36^ Despite this aspect, additional crystalline peaks are observed for AgNPs (curve in red) at 28.92^ο^ (210), 31.86^ο^ (113), and 45.84^ο^ (124),^33^ confirming the presence of the AgNPs. As expected, an amorphous behavior is observed for CD, confirming the complete conversion of ciprofloxacin (elimination of the crystalline peaks from the precursor).

**Figure 2:**
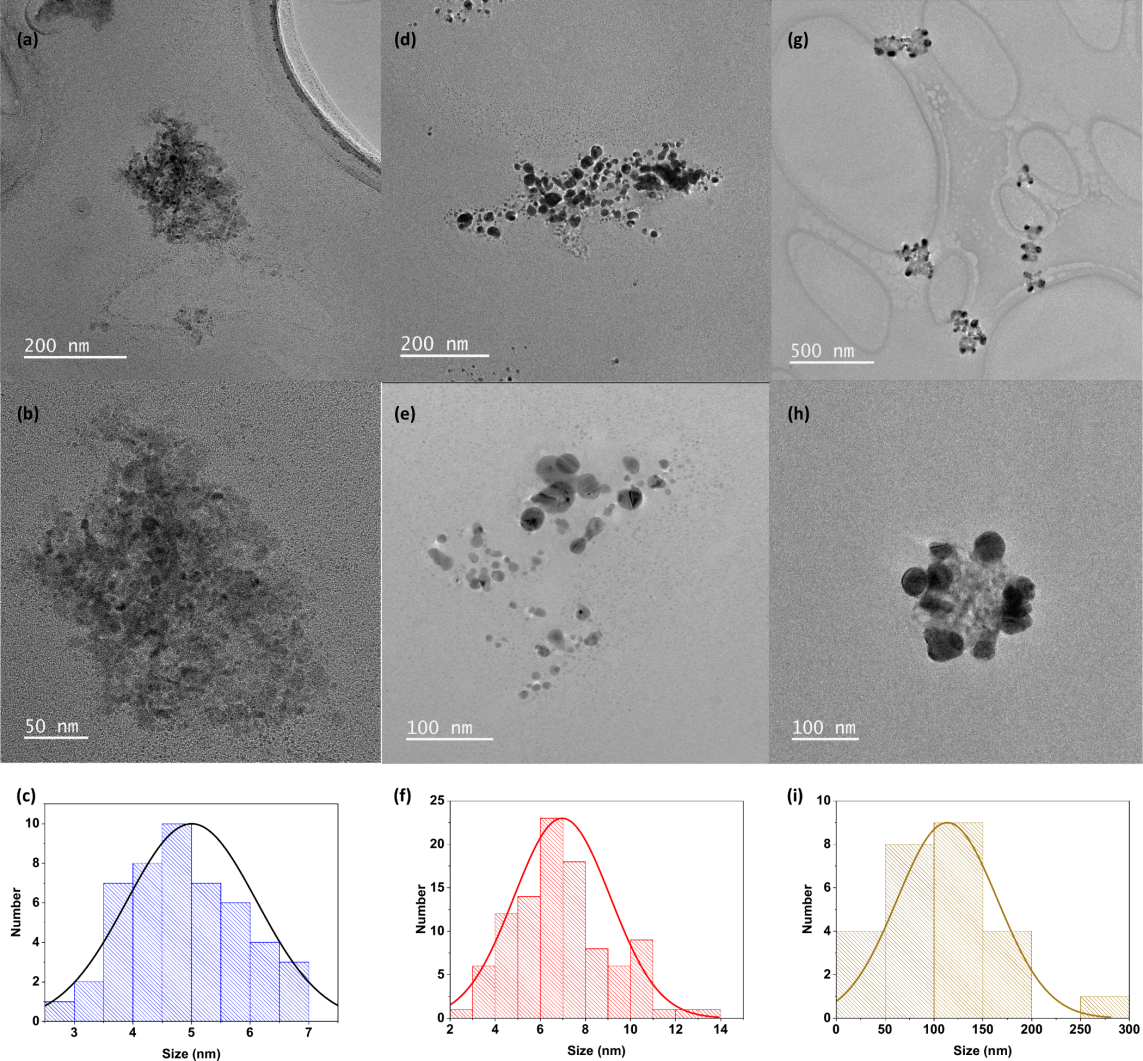
TEM images for CD nanoparticles at different magnification levels (a, b) and the corresponding distribution of size (c). TEM images for AgNPs at different magnification levels (d, e) and the corresponding distribution of size (f). TEM images for CD@AgNPs at different magnification levels (g, h) and the corresponding distribution of size (i).

TEM images were used to evaluate the morphology of the synthesized nanoparticles, as summarized in Figure 2. As shown in Figure 2a (with the magnified image in Figure 2b), aggregates of nanoparticles with the diameter centered at 5 nm were observed for pure CD nanoparticles disposed of as aggregates. The histogram in Figure 2c confirms a variation in CD size distribution from 3 to 7 nm. The morphology of AgNPs is shown in Figure 2d,e, with the distribution of size centered at 7 nm in an overall range of size distributions from 2 to 14 nm (see Figure 2f). For composites of AgNPs and CD (see Figure 2g,h), the observed structures are composed of aggregates of CDs surrounded by AgNP nanostructures forming clusters of CD@AgNPs with size distribution centered at 150 nm, as shown in Figure 2i. The disposition of these nanostructures in aggregates was also detected from dynamic scattering (DLS), which indicates a distribution of sizes centered at 295.3 nm, a polydispersity index (PDI) of 0.251 for CDs, and a size of particles of 119.3 nm for AgNPs (PDI = 0.253). Regarding the response of the composites of CD@AgNPs, the distribution of particles around 253 nm with a PDI of 0.557 indicates that composites are more polydisperse than the corresponding components (AgNPs and CDs).

For biological applications, important information is provided by Miao et al.,^25^ who evaluated the cytotoxicity analysis of CDs derived from ciprofloxacin with the study of the biocompatibility of 3T3 cells. The authors reported a cell survival above 80% after coincubation with CD derivatives in the range of 0–100 μg/mL for 24 h. Below the concentration of nanoparticles of 50 μg/mL, the system’s response returned to 100% in the survival rate of the cells, characterizing a negligible effect on cell growth. Regarding the evaluation of cytotoxicity against human cells, standard methyl thiazolyl tetrazolium assays were considered to identify the cytotoxicity in human epithelial cells (HeLa cells) using different capping agents for AgNPs and light excitation conditions for CDs. It is generally reported as a dose-dependent phenomenon in AgNP-loaded alginate, with a good biocompatibility degree (higher cell viability) for regular cell lines.^37,38^ On the other hand, lower cell viability is observed for HeLa cancerous cells, characterizing applications for Ag-based systems.^37,39,40^ Also, additional encapsulation of sodium alginate AgNPs in poly(vinyl alcohol) exhibited noncytotoxicity against human skin fibroblasts,^37^ which is in agreement with tests reported for AgNP-doped hydroxyapatite/alginate nanoparticles.^41^ For antibiotic-based CDs in plasmonic nanohybrid compounds,^42^ two critical aspects must be considered in the overall cytotoxicity: the Ag-based dose-dependent response and the effect of light on CD activity. While low toxicity is observed for CDs in the absence of light (viability >90%), under exposure to light, the viability is reduced to values in the order of 85%, as reported in ref (43), as a consequence of the photodegradation of CDs.^44^

Based on this information, the evaluation of the antibacterial activity of the synthesized nanoparticles (CD, AgNPs, and CD@AgNPs) was performed from MIC and MBC assays. These experiments considered successive dilutions of nanoparticle solutions from the mother solution of the as-prepared nanoparticles (viz. *C*_0_ for converted CDs from an initial concentration of 10 mg/mL of the ciprofloxacin (the precursor) and *C*_1_ for the mother solution of the AgNPs) in which each experimental system inhibits *S. aureus* and *E. coli*. The MBC (MIC) values obtained against *S. aureus* were , , and  relative to systems CD, AgNPs, and CD@AgNPs, respectively, indicating the best performance against *S. aureus* for the association of CD@AgNPs due to the higher dilution degree relative to the initial concentration of the mother solution. On the other hand, for treatment against *E. coli*, the MBC (MIC) values were , , and  relative to system CD, AgNPs, and CD@AgNPs, respectively, confirming the best performance for the association of CD and AgNPs observed for both bacteria and the advantage relative to the lower MBC/MIC values against the Gram-negative bacteria.

A comparison with results (MBC/MIC) for the corresponding experimental systems reported in the literature is provided in Table 1.

**Table 1:** Values for MIC and MBC for Green AgNPs, CDs, and Conventional Antibiotics against *S. aureus* and *E. coli*

active agent	source/reducer	strain	MIC	MBC	ref
AgNPs	Equisetum diffusum extract	*E. coli*	100 μg/mL		(45)
		ATCC19115			
AgNPs	AgNO_3_/PSBA	*S. aureus*	3.5 μg/mL		(46)
		*E. coli*	9.1 μg/mL		
		*B. subtilis*	6 μg/mL		
		*S. typhi* (clinically isolated)	23.3 μg/mL		
AgNPs	AgNO_3_ /NaBH_4_	*S. aureus*	5.1 μg/mL		(47)
		*E. coli*	13.7 μg/mL		
		*B. subtilis*	4.5 μg/mL		
		*S. typhi* (clinically isolated)	16.2 μg/mL		
AuNPs	*Fagonia arabica* leaves	*C. sakazakii* and L. monocytogenes	62 μg/mL		(48)
			25 μg/mL		
CD	lemon peel	*S. aureus*	100 μg/μL	50 μg/mL	(49)
CD	protamine sulfate	*S. aureus* (CCTCC AB 91093)	25 μg/mL		(50)
		*E. coli* (CCTCC AB 93154)	2000 μg/mL		
CD	turmeric leaf	*S. aureus* (ATCC 29213)	0.25 mg/mL		(51)
		*E. coli* (ATCC 25922)	0.25 mg/mL		
CD	arginine	*S. aureus* (ATCC 6538*)*	6.25 mg/mL	12.5 mg/mL	(52)
		*E. coli* (ATCC 8739*)*	6.25 mg/mL	12.5 mg/mL	
CD	honey	*S. aureus*	1.8 mg/mL	1.8 mg/mL	(53)
		*E. coli*	1.8 mg/mL	1.8 mg/mL	
CD	epigynum auritum leaves	*S. aureus*	8 μg/mL	32 μg/mL	(54)
		*E. coli*	62 μg/mL	124 μg/mL	
CD	*Cupressus lusitanica*	*S. aureus*	500 μg/mL	500 μg/mL	(55)
		*E. coli*	250 μg/mL	250 μg/mL	
AgNPs	citral-tryptamine	*S. aureus*	15.00 μg/mL		(56)
		*E. coli*	11.30 μg/mL		
AgNPs	Argovit-C	*S. aureus* (ATCC 25953*)*	2.90 μg/mL	3.9 μg/mL	(57)
		*E. coli* (ATCC 25922*)*	0.95 μg/mL	2.0 μg/mL	
AgNPs	chemical synthesis	*S. aureus* (ATCC 25923*)*	4.0 μg/mL	8.0 μg/mL	(58)
		*E. coli* (ATCC 25922*)*	2.0 μg/mL	4.0 μg/mL	
AgNPs	ferulic acid + lignin	*S. aureus*	25.0 μg/mL		(59)
		*E. coli*	12.5 μg/mL		
AgNPs	chemical synthesis	*S. aureus*	16 μg/mL		(60)
		*E. coli*	2 μg/mL		
AgNPs	Blumea balsamiferaoil	*S. aureus* (ATCC 25923)	0.023 mg/mL	0.05 mg/mL	(61)
		*E. coli* (PV 1393)	0.012 mg/mL	0.023 mg/mL	
AgNPs	Ginkgo biloba leaves	*S. aureus* (ATCC 25923)	8.0 μg/mL	32 μg/mL	(62)
		*E. coli* (ATCC 25922)	4.0 μg/mL	8 μg/mL	
AgNPs	*Clematis gouriana*	*S. aureus*	6.25 μg/mL	13.5 μg/mL	(63)
		*E. coli*	13.5 μg/mL	26 μg/mL	
AgNPs	*Fusarium oxysporum* enzymes	*S. aureus* ( ATCC 25923*)*	16.98 μg/mL	16.98 μg/mL	(64)
		*E. coli* (ATCC 25922)	4.24 μg/mL	4.24 μg/mL	
AgNPs	ficus deltoidea Jack leaf	*S. aureus* (ATCC 6538)	2.50 mg/mL	5.00 mg/mL	(65)
		*E. coli* (ATCC 11229)	2.50 mg/mL	5.00 mg/mL	
CD	ciprofloxacin	*S. aureus*	1:16	1:32	this work
		*E. coli*	1:128	1:128	
AgNPs	sodium alginate	*S. aureus*	1:2	1:2	this work
		*E. coli*	1:4	1:4	
CD@AgNPs	ciprofloxacin/sodium alginate	*S. aureus*	1:64	1:64	this work
		*E. coli*	1:512	1:512	
ampicillin		*S. aureus* (clinical isolate)	0.5 μg/mL		(66)
		*E. coli* DH5α (nonpathogenic)	2.0 μg/mL		
tetracycline		*S. aureus* (ATCC 25923)	0.97 μg/mL		(67)
		*E. coli* (ATCC 25922)	0.97 μg/mL		
vancomycin		*S. aureus* (ATCC 12600)	16.0 μg/mL	128.0 μg/mL	(68)
		*E. coli* (ATCC 9637)	128.0 μg/mL	256.0 μg/mL	
amoxicillin		*S. aureus* (ATCC 25922)	0.5 μg/mL	1 μg/mL	(69)
ciprofloxacin		*S. aureus* (ATCC 25923)	0.24 μg/mL		(70)
ciprofloxacin		*E. coli* (ATCC 25922)	0.006 μg/mL	0.006 μg/mL	(71)

**Figure 3:**
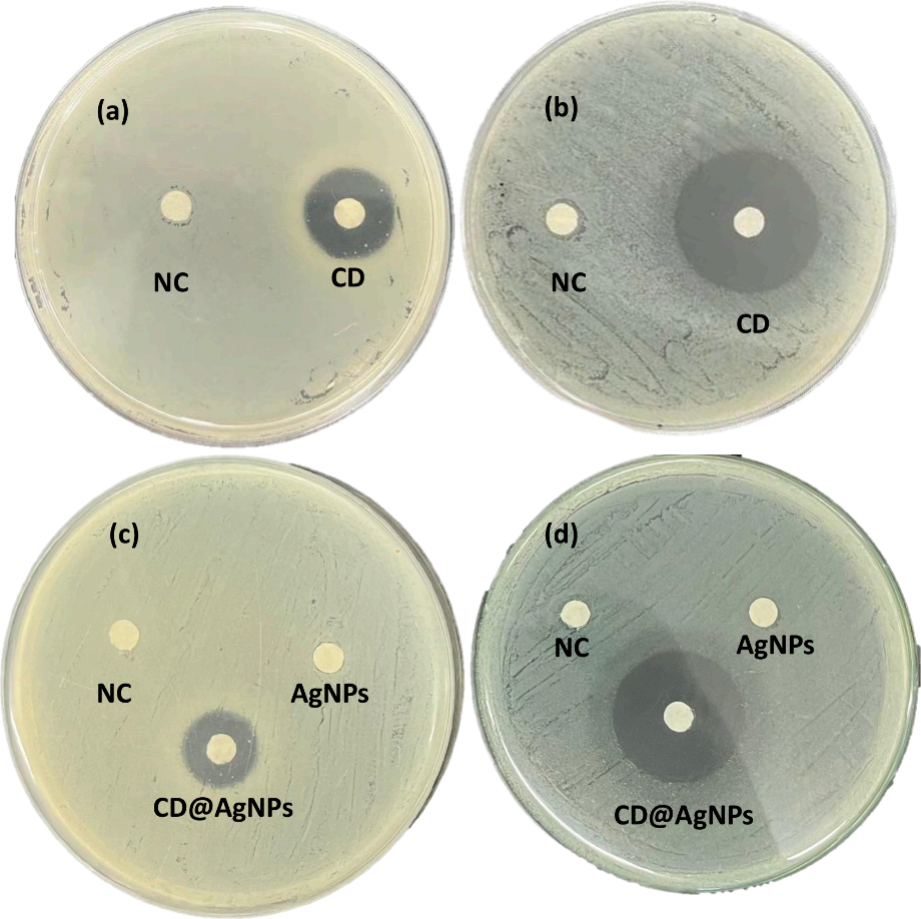
Inhibition halo measurement for disks impregnated with CD, CD@AgNPs, and AgNPs in comparison with a control disk in plates containing *S. aureus* (a) and (c) and *E. coli* (b) and (d).

As can be observed from Table 1, a typical increase in the concentration of CDs and AgNPs relative to MIC/MBC values in comparison with standard antibiotics is detected—while conventional antibiotics typically have values in the order of μg/mL, a broad range of values from μg/mL to mg/mL is observed for green synthesized materials, indicating that the reducing agent plays a critical role in the overall performance of the resulting nanostructures. In addition to MBC/MIC determination, halo diffusion assays were conducted (results are shown in Figure 3a,c for *S. aureus* and in Figure 3b,d for *E. coli*). Figure 3a shows a negligible inhibition halo for the negative control (pure filter paper). In contrast, a halo of 19 mm is observed for CD nanoparticles against *S. aureus*, while an inhibition halo of 31 mm is observed against *E. coli*. The response of CD@AgNPs against *S. aureus* shows an inhibition halo of 20 mm, while the corresponding value against *E. coli* is 33 mm.

**Figure 4:**
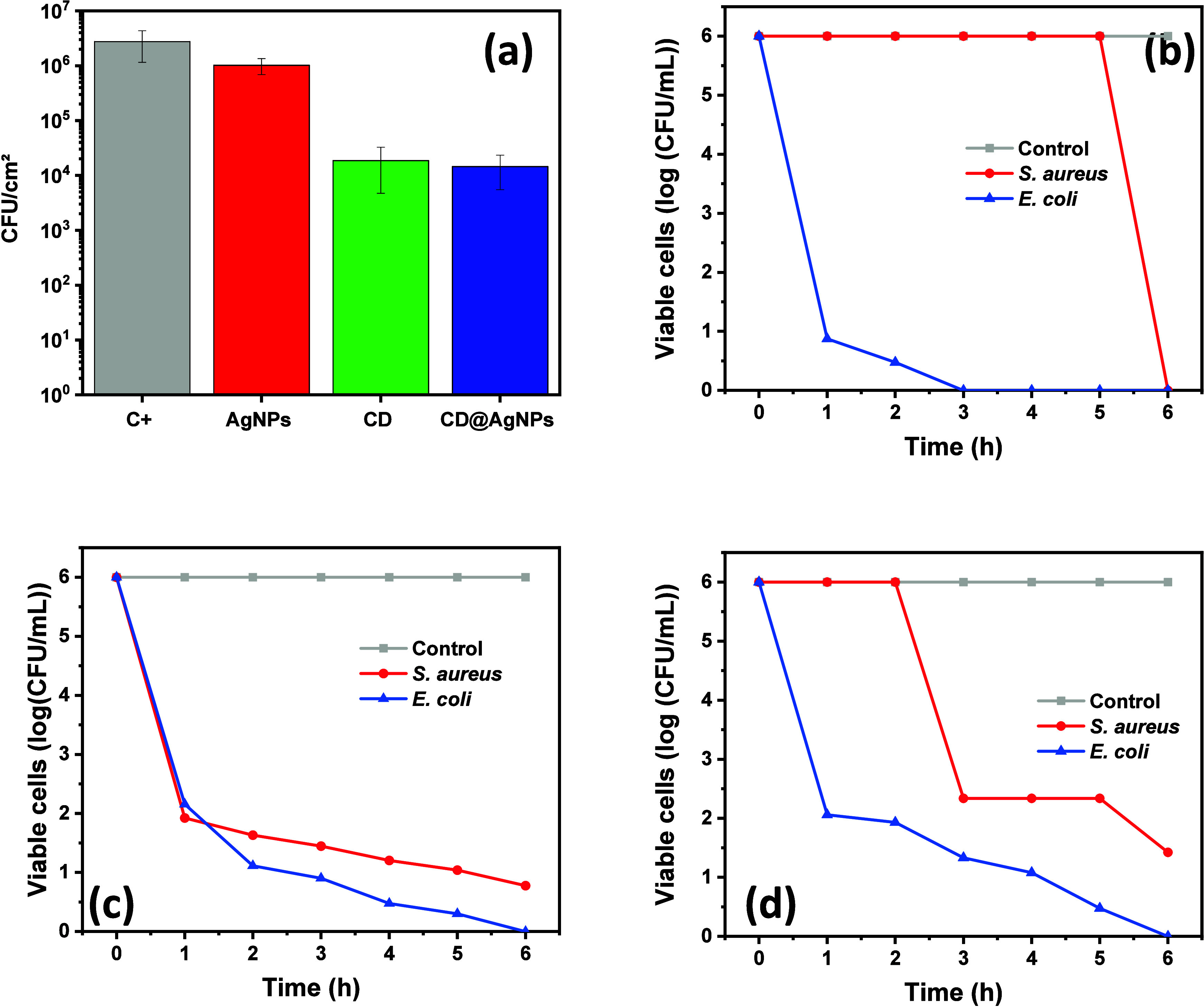
(a) Viable cell counting of the consolidated biofilm of *S. aureus* after treatments with CD and CD@AgNPs compared with the negative control. (b) Viable cell counting of *S. aureus* and *E. coli* as a function of treatment time with CD compared with the negative control. (c) Viable cell counting of *S. aureus* and *E. coli* as a function of treatment time with AgNPs compared with the negative control. (d) Viable cell counting of *S. aureus* and *E. coli* as a function of treatment time with CD@AgNPs compared with the negative control.

For antibiofilm assays,^72^ the experimental systems were assessed regarding the biofilm eradication test (*S. aureus* strains). The CD-based system reduced the biofilm formation by 99.32% compared to the positive control, while CD@AgNPs exhibited a slight improvement (99.47%) against biofilms consolidated for 24 h. For comparison, the performance of AgNPs was 62.88%, as shown in Figure 4a.

The kinetics of bacterial inhibition from nanomaterials were evaluated by considering time-kill assays. The antibacterial treatment takes place at a controlled contact time, followed by incubation, and after that, the viable cell density is counted. As expected, the number of viable cells (under negative control) proved to have negligible variation in all of the experiments.

On the other hand, the number of viable cells against the system CD indicates a substantial reduction after 5 h of interaction with *S. aureus* (see Figure 4b). In agreement with previously reported experiments, a more robust and faster activity is observed against *E. coli*, significantly reducing the number of viable cells after the first hour of treatment (a 5-log reduction in colony-forming units (CFU/mL)). For bacteria treated with AgNPs, a high slope variation is observed in the first hour of treatment, followed by a linear decrease in the viable cells under treatment with AgNPs (Figure 4c). In agreement with the previously reported data, the plot in Figure 4d indicates an improved performance of the system CD@AgNPs against *E. coli* in comparison with the treatment against *S. aureus*, from which it is possible to observe a general decrease in the induction time of the antibacterial activity (1 h for *E. coli* and 3 h for *S. aureus*).

The data from characterization techniques confirmed the effective conversion of fluoroquinolone derivatives into CDs by their amorphous signature in the DRX pattern of nanoparticles and the high quantum yield fluorescence. As observed, composites of CD@AgNPs showed superior antibacterial performance compared with pure CDs and AgNPs, with the prevailing activity attributed to CDs.

The general mechanism for the antibacterial activity of CD-based compounds can be attributed to the cationic effect of nanoparticles: negative surface charge is observed in both Gram-positive (due to the lipoteichoic acid) and Gram-negative species (due to the liposaccharides). Consequently, positively charged species (CD@AgNPs) are electrostatically attracted to the target (bacterial cell walls) direction, facilitating the adhesion, loss of electrolytes, and the release of Ag^+^ ions inside the bacteria.^73^

The ions can interrupt vital processes for bacteria survival, leading to ROS production in cellular components.^74^ The more effective antibacterial activity against *E. coli* than *S. aureus* can be attributed to different factors: the thicker peptidoglycan layer of *S. aureus* introduces an extra barrier for the interaction of CDs and the inner part of the bacterial cells. On the other hand, the effective antibacterial activity of the composites against *E. coli* and *S. aureus* strains can be attributed to the even more negative ZP for Gram-negative than Gram-positive bacteria due to the negatively charged LPS layer in the first group^75^ that interacts strongly with the positively charged species (CD and CD@AgNPs).

## Conclusions

The highly fluorescent species derived from ciprofloxacin proved to be an essential support for association with AgNPs, presenting intrinsic antibacterial and antibiofilm activity against *S. aureus* and *E. coli*. The positive charge present on the surface of CDs (pure and in association with AgNPs) favors the stability of nanoparticles in solution, allowing the promising future application as sanitizing agents or also as wound dressing agents under adequate disposition of nanoparticles on adsorbent surfaces loaded with CD-based nanostructures. The electrostatic interaction between CDs and bacterial cells results in the attachment and the subsequent rupture/penetration of the cell wall, making possible the activity of ROS generated by AgNPs and CDs, resulting in the solid antibacterial activity for the produced nanostructures.
